# Covert Shift of Attention Modulates the Ongoing Neural Activity in a Reaching Area of the Macaque Dorsomedial Visual Stream

**DOI:** 10.1371/journal.pone.0015078

**Published:** 2010-11-29

**Authors:** Claudio Galletti, Rossella Breveglieri, Markus Lappe, Annalisa Bosco, Marco Ciavarro, Patrizia Fattori

**Affiliations:** 1 Dipartimento di Fisiologia Umana e Generale, Universita' di Bologna, Bologna, Italy; 2 Department of Psychology and Otto Creutzfeldt Center for Cognitive and Behavioral Neuroscience, Westfälische Wilhelms-University, Münster, Germany; Rutgers University, United States of America

## Abstract

**Background:**

Attention is used to enhance neural processing of selected parts of a visual scene. It increases neural responses to stimuli near target locations and is usually coupled to eye movements. Covert attention shifts, however, decouple the attentional focus from gaze, allowing to direct the attention to a peripheral location without moving the eyes. We tested whether covert attention shifts modulate ongoing neuronal activity in cortical area V6A, an area that provides a bridge between visual signals and arm-motor control.

**Methodology/Principal Findings:**

We performed single cell recordings from 3 Macaca Fascicularis trained to fixate straight-head, while shifting attention outward to a peripheral cue and inward again to the fixation point. We found that neurons in V6A are influenced by spatial attention. The attentional modulation occurs without gaze shifts and cannot be explained by visual stimulations. Visual, motor, and attentional responses can occur in combination in single neurons.

**Conclusions/Significance:**

This modulation in an area primarily involved in visuo-motor transformation for reaching may form a neural basis for coupling attention to the preparation of reaching movements. Our results show that cortical processes of attention are related not only to eye-movements, as many studies have shown, but also to arm movements, a finding that has been suggested by some previous behavioral findings. Therefore, the widely-held view that spatial attention is tightly intertwined with—and perhaps directly derived from—motor preparatory processes should be extended to a broader spectrum of motor processes than just eye movements.

## Introduction

When we want to recognize an object in the field of view, or want to grasp it, we typically direct our gaze towards the object. The shift of gaze is the consequence, and the overt evidence as well, of the shift of our attention towards the object of interest. Although under normal circumstances the direction of attention and the direction of gaze are aligned, we are able to disengage attention from the point of fixation. This ability, known as covert spatial attention, allows us to select and acquire peripheral visual information without shifting gaze [1], [2].

Attention enhances both behavioral and neuronal performances [3]. Reaction to attended targets is faster than to unattended targets [1], and responses of neurons to covertly attended stimuli are enhanced relative to those of unattended stimuli [4], [5], [see 6 for a review], [7], [8]. Thus, attention modulates the processing of information in visual cortical maps, and selects parts of the scene to receive increased processing resources.

The selection of the part of the scene to receive attention, i.e. the control of the focus of attention, is driven by the saliency of the stimuli and by the requirements of the task that is currently performed. If motor actions are to be performed on the selected targets, the focus of attention is closely related to these actions. The initiation of a saccade, for instance, is preceded by a mandatory shift of attention towards the saccade goal [9], [10], [11], [12]. The deployment of attention is linked to the mechanisms of selecting a saccade target and preparing the saccade even for covert attention shifts [13], [14], [15], [16], [17], [but see also 18].

The link between attention and goal-directed motor action is not confined to eye movements. Also the preparation of reaching movements is paralleled by a shift of attention to the goal of the reach [19], [20]. Therefore, one might expect that, similar to oculomotor areas that provide signals for overt and covert shifts of attention, also cortical areas that are involved in arm movements may contribute to shifts of attention, or may use spatial attentional signals to prepare arm movement or direct the hand towards the object to be grasped.

The medial posterior-parietal area V6A acts as a bridge between visual processing and arm motor coding [21]. Our aim in this work was to find out whether the activity of single cells in V6A is influenced by shifts of covert attention. Since, usually, the direction of gaze and the direction of attention are aligned, and since area V6A contains a high percentage of gaze-dependent neurons [22], we had to disengage attention from the point of fixation (covert attention) in order to demonstrate that the direction of attention, and not the direction of gaze, modulates V6A neurons. In a task specifically designed for this, we found that the neural modulation was still present when covert attention was shifted without any concurrent shift of the direction of gaze. We suggest that this attentional modulation is helpful in guiding the hand during reach-to-grasp movements, particularly when the movements are directed towards non-foveated objects.

## Results

We performed extracellular recordings on 182 single cells of area V6A in 3 Macaca fascicularis. Cells were ascribed to V6A following the functional criteria described in Galletti et al. [23], and on cytoarchitectonic criteria according to Luppino et al. [24].

**Figure 1:**
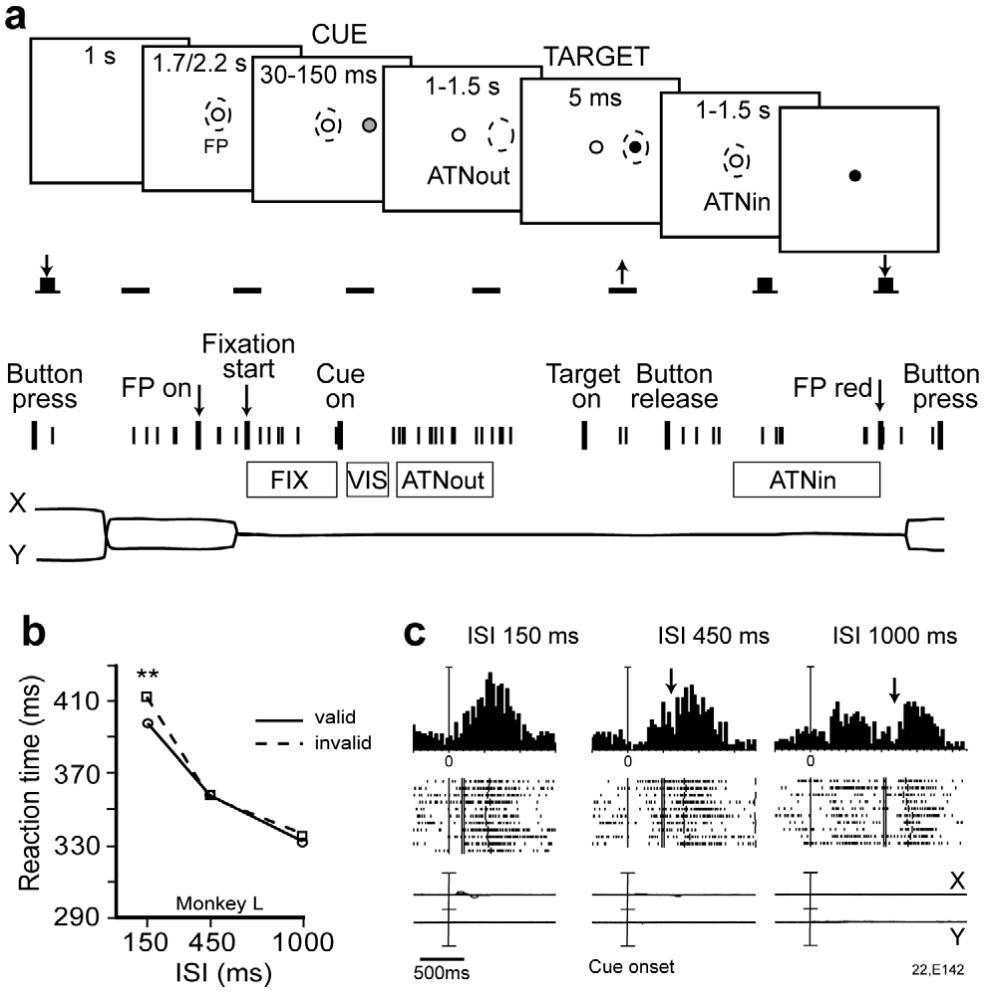
Attentional task and effects in V6A. **a**) Schematic representation of the task. Top: Sequence of events in a single trial. After button pressing, the monkey maintained fixation on the central fixation point (white dot, FP) all throughout the trial while covertly shifting attention (dashed circle) towards the cued location (grey dot). After target (black dot) detection, the animal released the button, continuing to gaze the fixation point until it changed in color (from green to red). Color-change detection was reported by the animal by button pressing. Bottom: typical example of neural activity and eye traces during a single trial. Short vertical ticks are spikes. Long vertical ticks among spikes indicate the occurrence of behavioral events (markers). Below the neural trace, time epochs during a typical trial are indicated. FIX: fixation epoch, VIS: visual epoch, ATNout: outward attention epoch, ATNin: inward attention epoch. **b**) Performance of 1 monkey expressed as reaction time to detect the target at different inter-stimulus-intervals (ISIs). Results from valid (continuous) and invalid (dashed) trials are shown. Significant difference in reaction times between valid and invalid trials at ISI 150 shows that attention is directed towards the peripheral cue location at this time. **c**) Peri-stimulus time histograms of an example neuron recorded with different ISIs. Trials are aligned to cue onset. The neuron shows two discharges (after cue onset and button release, respectively) that separate (arrow) clearly at longer ISIs.

Animals were trained to fixate a light-emitting diode (LED) in the straight-ahead position in darkness while pressing a button located outside their field of view. While fixating, the monkeys had to detect a target (5 ms red flash) in one out of several peripheral positions and respond to it by releasing the button without moving the eyes (Fig. 1**a**). The target position was cued by a yellow flash (30–150 ms) preceding the target onset by 1–1.5 s. The cue signal prompted the monkeys to covertly displace attention towards the periphery. After target detection, the monkeys shifted attention back towards the straight-ahead position to detect the change in color of the fixation LED. This change in color had to be reported by pressing the button again. The monkeys were trained to maintain gaze in the straight-ahead position all throughout the trial. Their fixation was checked using an electronic window (5°×5°) and off line inspection of recorded eye traces.

We quantified each cell's discharge during three time epochs (see Fig. 1**a**): the starting fixation epoch before cue onset (baseline activity, FIX), the epoch from 200 to 500 ms after cue onset (covert attention shifted towards the cue location, ‘outward attention’), and the epoch from 400 ms after button release to the change in color of the fixation LED, when attention is again directed towards the central fixation point (‘inward attention’). We also analyzed passive visual response to the cue appearance in an epoch from 40 to 150 ms after the cue onset (VIS).

### Behavioral bases of covert attention shift

To check whether our experimental conditions induced covert attention shifts, we measured reaction times (RTs) between target onset and button release in one monkey. These measurements were collected in separate behavioral testing sessions before the onset of single unit recording. These sessions contained valid trials as described above, and invalid trials in which the cue was misleading because the target appeared on the opposite side. It is well known that effects of covert attention shifts are reflected in differences in the reaction times between valid and invalid trials both in human [1] and monkey [25]. In valid trials, especially with brief inter-stimulus-interval (ISI), the reaction time are expected to be shorter than during invalid trials because the location where the target appears benefits from attentional enhancement evoked by cue appearance.

As reported in Figure 1b, reaction times for target detection in valid and invalid trials were recorded at ISIs of 150, 450 and 1000 ms (Monkey L). Mean reaction times were 400.01 ms (ISI 150), 360.01 ms (ISI 450) and 335.90 ms (ISI 1000) for valid trials, and 412.89 ms (ISI 150), 357.35 ms (ISI 450) and 336.16 ms (ISI 1000) for invalid trials. These data were entered in 3x2 repeated measures ANOVA with ISI (150, 450 and 1000) and validity (Valid vs invalid trials) as within factors. The ANOVA has revealed a significant interaction ISI x validity (F(2,36) = 5.47, p = 0.008) with a difference in reaction time between valid and invalid trials occurred for the ISI of 150 ms (p = 0.0009, Newman-Keuls post hoc test). The shorter RT for valid trials is an indicator of attention allocated to the cue, and confirms that the experimental paradigm we used elicited covert attention shifts in our monkey subjects. For longer ISIs, the validity effect was no longer significant, although reaction time for both trial types decreased with increasing ISI (repeated measures ANOVA, main effect of factor ISI, F(2,36) = 72.87, p = 0.000001) suggesting an increase of alertness when the ISI is longer.

### Single-unit recordings

Since significant RT difference between valid and invalid trials was observed for ISI of 150 ms but not for ISIs of 450 ms and higher, and because we wanted to exclude from the analysis the effect of putative visual responses to cue onset, we restricted the analysis of the effect of outward attention shifts to a time epoch from 200 and 500 ms after cue appearance. However, we performed also the analysis with a time window from 150 ms to 450 ms and the results were the same. Below, we report the results of the former analysis as a more conservative approach.

Since key-press and key-release actions elicited neural responses in V6A (Galletti et al., 1997; Marzocchi et al., 2008), we wanted to separate in time the responses related to outward shifts of attention from the responses related to the button press. In preliminary experiments we varied ISI during cell recordings in order to find a timing in which the attentional responses are clearly separable from the button release responses. Figure 1**c** shows an example of a cell recorded with different ISIs (150, 450 and 1000 ms, tested in randomly interleaved trials) and a cue duration of 30 ms. When the ISI was 150 ms (Fig. 1**c** left), the cell had a strong and long discharge starting immediately after the cue onset. An increase of the ISI to 450 ms (Fig. 1**c**, center) caused the tendency of the discharge to separate in 2 components (see arrow in Fig. 1**c**, center). These two components became further separated and distinguishable at an ISI of 1000 ms (see arrow in Fig. 1**c**, right), the first component related to the cue, the second to the button release. The first component contains the effect of exogneous attention following the presentation of the cue and appears at all ISIs, even for long ISIs of 1000 ms. Therefore, although the behavioral effect of exogenous attention on the reaction time to the cue wears off after long ISIs (Fig. 1b), the transient physiological effect on the cell response between 200 and 500 ms is measurable at all ISIs., Since the cue response component was clearly separable from the button release component only at an ISI longer than 450 ms, we used ISIs of 1000 and 1500 ms for all subsequent neural recordings.

Of 182 recorded cells, 83 (46%) showed neural discharges during the outward and/or inward attention epochs that were significantly different from the baseline (epoch FIX) as assessed by Student's t-test (with Bonferroni correction, p<0.02). From now on, we will refer to these cells as ‘task-related cells’.

### Neural responses during outward attention

Fifty-one task-related cells were modulated during outward attention epoch (Student' t-test, p<0.05). In particular, 24 cells (47%) were inhibited (i. e. the discharge during outward attention epoch was weaker than during FIX), and 27 cells (53%) were excited (i. e. the discharge during outward attention epoch was stronger than during FIX).

**Figure 2:**
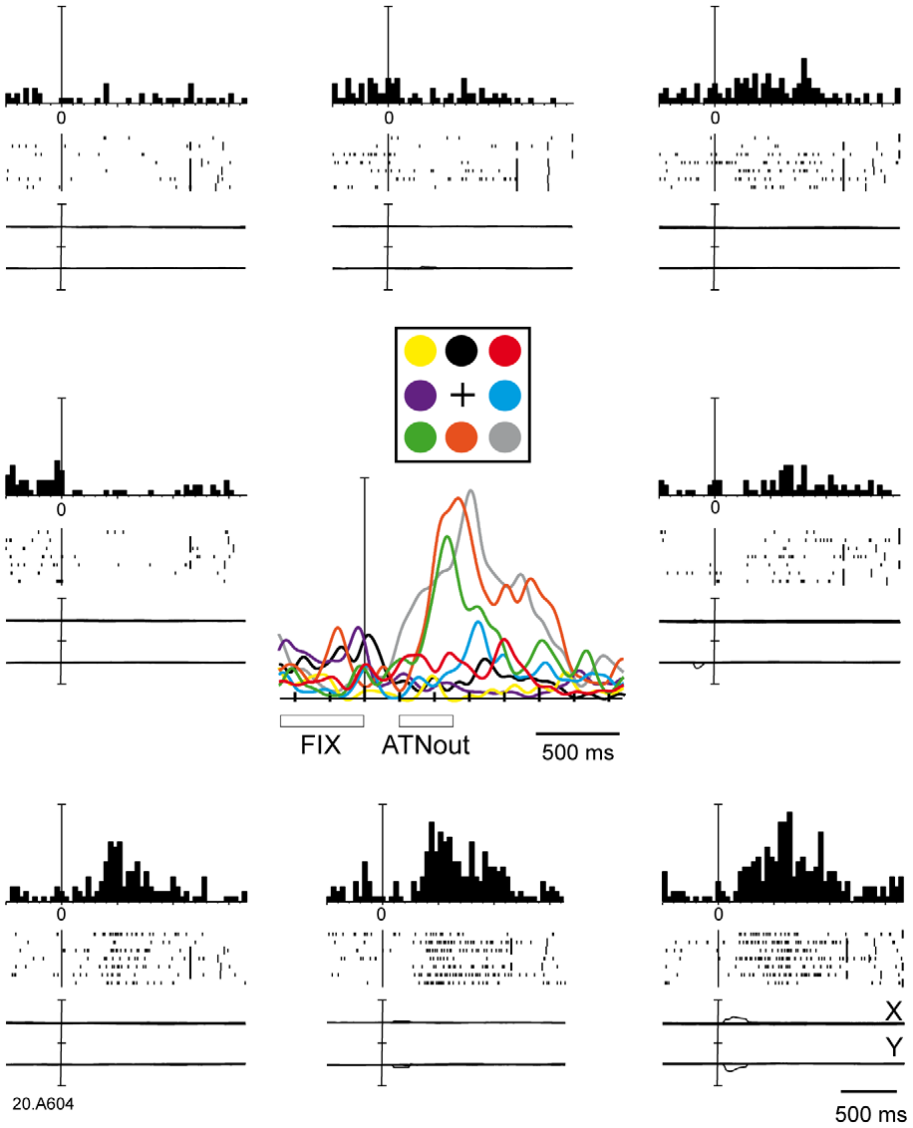
Example of spatially-tuned modulations of neural activity during outward attention epoch. The neuron shows a strong discharge during outward attention epoch preferring covert shifts of attention towards the bottom part of the space. Each inset contains the peri-event time histogram, raster plots and eye position signals, and is positioned in the same relative position as the cue on the panel. In the central part of the figure, the spike density functions (SDFs) of the activity for each of the 8 cue positions are superimposed and aligned on the cue onset. The mean duration of epochs FIX and outward attention is indicated below the SDFs. Neural activity and eye traces are aligned on the cue onset. Scalebar in peri-event time histograms, 70 spikes/s. Binwidth, 40 ms. Eyetraces: scalebar, 60°. Other details as in Figure 1.

Figure 2 shows a cell with a typical outward attention response for cues presented in the lower space. The spatially-tuned outward attention activity had a very long latency (on average 283 ms). The cell discharged strongly after cue onset and continued to discharge well after cue offset. In some trials, the response lasted until target onset, that is 1 s or more later than cue onset. This discharge was very different from a typical V6A visual response [23] To compare the effect of what we call 'outward attention' to a purely visual response in our neuronal sample we assessed the influence of the visual stimulation by the cue appearance (epoch VIS) on the firing rates. Consistent with earlier observations that a stationary light stimulus like the cue is not the most effective stimulus for V6A visual cells [23], only 40% of the cells (72/182) were modulated during VIS with respect to the baseline epoch FIX (Student' t-test, p<0.05).

**Figure 3:**
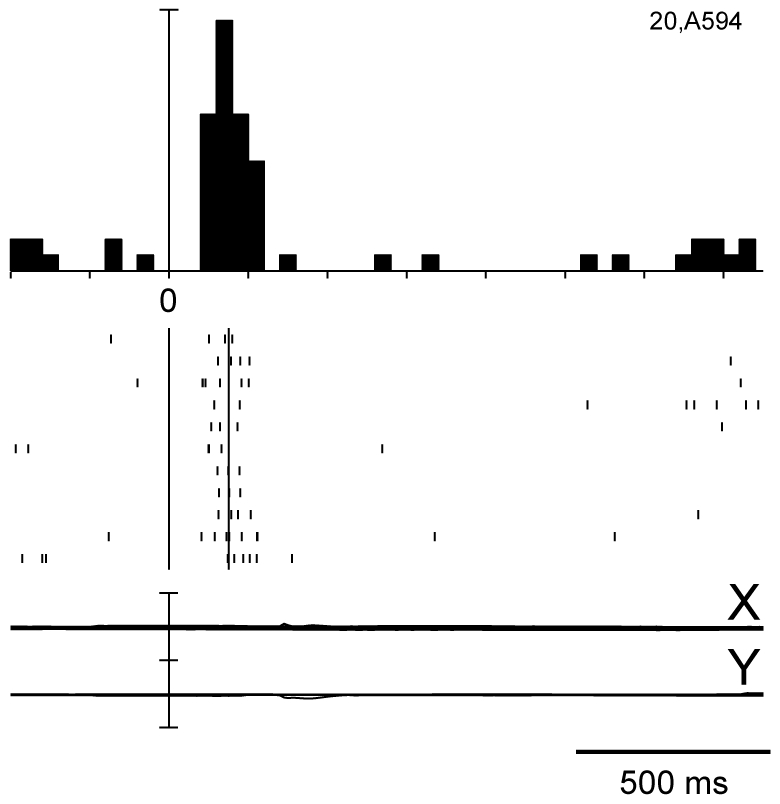
Typical visual response in V6A. Neural activity and eye traces are aligned with cue onset. Peri-event time histograms: binwidth, 40 ms; scalebars, 38 spikes/s. Eyetraces: scalebar, 60°. Other details as in figures 1 and 2.The response started about 80 ms after the cue onset. The cell showed a brisk response whose duration was similar to the duration of the stimulus (150 ms).

One example of a cell with a typical visual response to cue onset is shown in Figure 3. The response started about 80 ms after the cue onset. The cell showed a brisk response whose duration was similar to the duration of the stimulus (150 ms).

Comparing the discharges after cue presentation in Fig. 2 and 3, it is evident that the duration of the outward attention response was much longer than the visual stimulus, contrary to what happens in typical visual responses where stimulus and response durations are nearly the same. Second, the latency of outward attention response was much longer and less strictly time locked than the latency of a typical visual response.

Spatial tuning of the outward attention activity was a common finding in our sample of V6A neurons: twenty-six out of 51 cells (51%) resulted significantly spatially tuned (one-way ANOVA, p<0.05).

**Figure 4:**
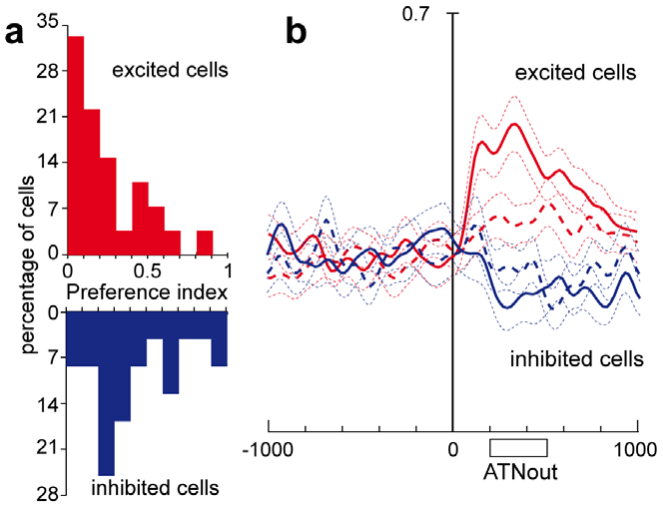
Activity modulation during outward attention epoch. **a**) Distribution of preference index (see Experimental procedures) for cells excited (red histogram) and inhibited (blue histogram) during outward attention epoch. **b**) Effect of the covert dislocation of the spotlight of attention on the activity of V6A cells during outward attention epoch. The average SDF for the excited (red lines) and inhibited (blue lines) cells are shown. Continuous lines represent the average SDF for the cue location evoking the maximal (excited cells) or minimal (inhibited cells) activity, and the dashed line that for the opposite location. Two dotted lines for each SDF indicate the variability band (SEM). The activity of cells in each population is aligned on the cue onset. Scale in abscissa: 200 ms/division; vertical scale 0.7. Other details as in Figure 1.

To investigate the direction sensitivity of cells with outward attention activity, we computed a preference index (PI, see Experimental Procedures). Figure 4**a** shows, separately, the distributions of PIs for excited (red) and inhibited (blue) cells. About half of the excited cells were direction selective, with a PI higher than 0.2. Note that the cell shown in Figure 2, that was strongly direction-selective, had a PI of 0.44. The inhibited cells were even more sensitive to the direction of covert attention, showing higher number of cells with high preference index.

Figure 4**b** shows the population activity of V6A cells that were excited (red lines) or inhibited (blue lines) during the epoch of outward attention. The continuous lines represent the average mean activity of cells in trials in which the cue appeared in the position evoking the maximum (excited) or the minimum (inhibited) discharge rate. The dashed lines represents the average mean activity of the cells in trials in which the cue appeared in the opposite position. The plots have been aligned on cue onset.

The discrimination between two opposite spatial positions at population level began around 100 ms after cue onset and peaked around 300 ms (Fig. 4**b**). This agrees with the time course of the shift of the spotlight of attention as assessed from the behavioral data: a behavioral effect of attention at the cued location was detectable 150 ms after the cue onset and ceased within 450 ms after the cue onset. Also the rapid change of population activity just after cue onset reported in Figure 4**b** well agrees with the fact that the displacement of the spotlight of attention during outward attention epoch is exogenously driven by the cue.

**Figure 5:**
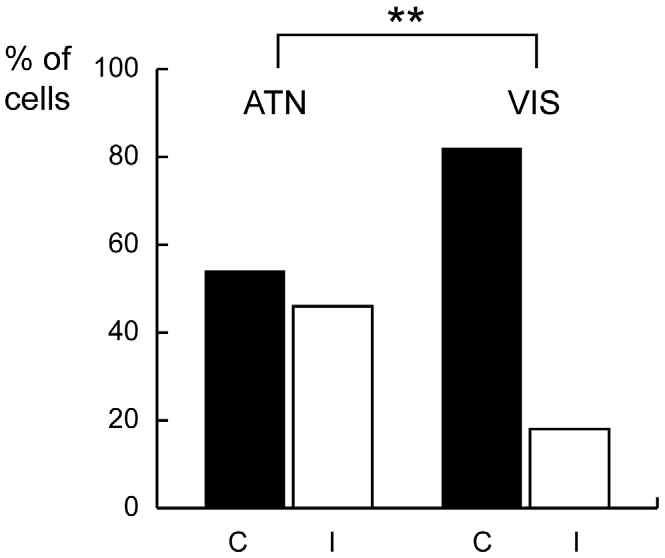
Preferred attentional and visual receptive-field locations in area V6A. Columns indicate the percentages of neurons modulated during outward attentional epoch (ATN) preferring contralateral (C) or ipsilateral (I) targets, and the percentages of visual cells (VIS) with the receptive-field center in the contralateral (C) or ipsilateral (I) hemifield. ATN and VIS populations include 26 and 684 cells, respectively. The percentage of visual cell with receptive fields centered in the contralateral hemifield was significantly higher than those centered in the ispilateral hemifield (Chi-squared test, chi-squared = 14.92, p<0.0001).

Independently from the effect of outward shift of attention (excitation or inhibition), the number of cells preferring contralateral shifts of covert attention (i.e. cells whose maximal discharge was for shifts towards parts of the space contralateral with respect to the recording site) was the same as that of cells preferring ipsilateral shifts (i.e. cells whose maximal discharge was for shifts towards parts of the space ipsilateral with respect to the recording site). Interestingly, the spatial distribution of visual receptive fields in V6A, mostly contralateral, is significantly different from the spatial selectivity of attentional responses (Chi-squared test, p<0.0001), as shown in Figure 5. This fact is against the view that the attentional effect could be the result of a modulation of the visual response, suggesting a functional separation between the two phenomena.

### Neural responses during inward attention

After target detection (i. e. after button release) the animal was requested to respond to a change in color of the fixation LED that occurred 1000 to 1500 ms after button release (see Fig. 1**a**). Thus, it is plausible that, during this period, the focus of attention was brought back to the fixation point (inward attention epoch). The fixation LED remained illuminated in the same color throughout the inward attention epoch, and no further visual stimulation was given after the target presentation and the button release. Since visual responses in area V6A are usually brief and brisk, the lasting modulations in the inward attention epoch cannot be ascribed only to the visual stimulation. They had to be related to endogenously driven shifts of attention towards the fixation point.

**Figure 6:**
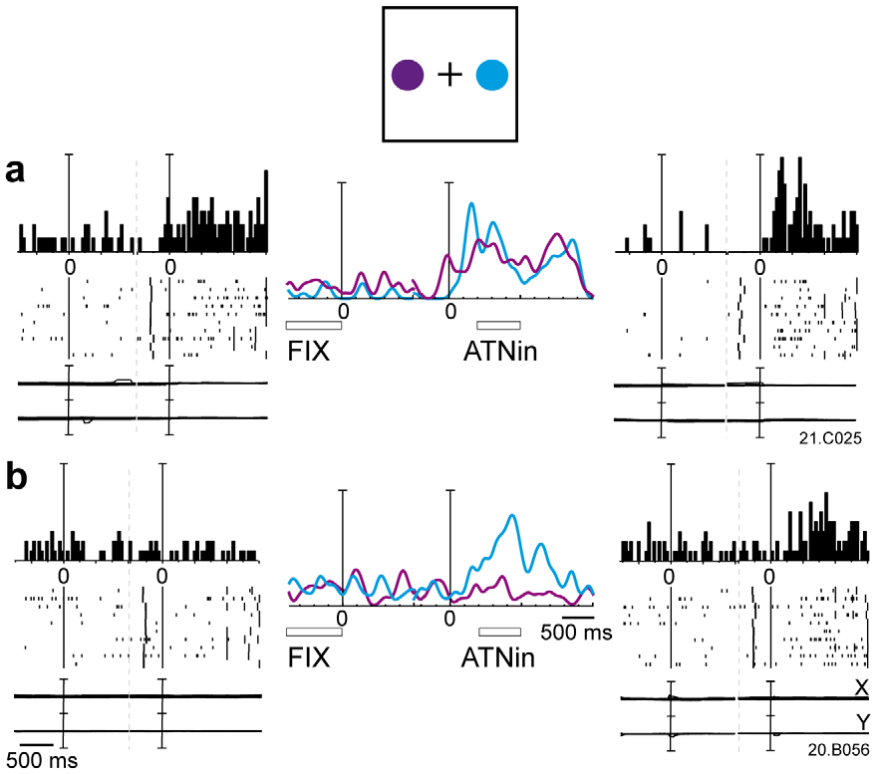
Examples of two neurons excited during inward attention epoch. **a**) Neuron excited during inward attention epoch, insensitive to the direction of the focus of attention. **b**) Neuron excited during inward attention epoch, sensitive to the direction of the focus of attention. Left and right: neural activity, raster dot displays and eye traces are aligned twice, with the cue onset (left) and with the button release (right). Center: SDFs of the two cue positions are superimposed (blue line: right position, purple line: left position). Peri-event time histograms: binwidth, 40 ms; scalebars, 18 spikes/s (**a**), 25 Spikes/s (**b**). Eyetraces: scalebar, 60°. Other details as in Figures 1 and 2.

Out of the task-related cells, 63 (76%) were significantly modulated during inward attention epoch with respect to the baseline (Student t-test, p<0.05): 33% of these cells were excited whereas the majority (67%) were inhibited. Figure 6**a** shows a cell with a strong discharge during inward attention epoch. This discharge occurred independently of the direction of covert attention during the preceding outward attention epoch (cue location). Most of the excited cells of our population showed this behavior (71%). Figure 6**b** shows a cell with direction selectivity: its response during inward attention epoch was different for the different cue positions. Neurons like these, showing a change in discharge in periods in which neither the processing of visual information, nor the execution of motor acts is taking place, strongly support the notion that attention modulates V6A neurons.

**Figure 7:**
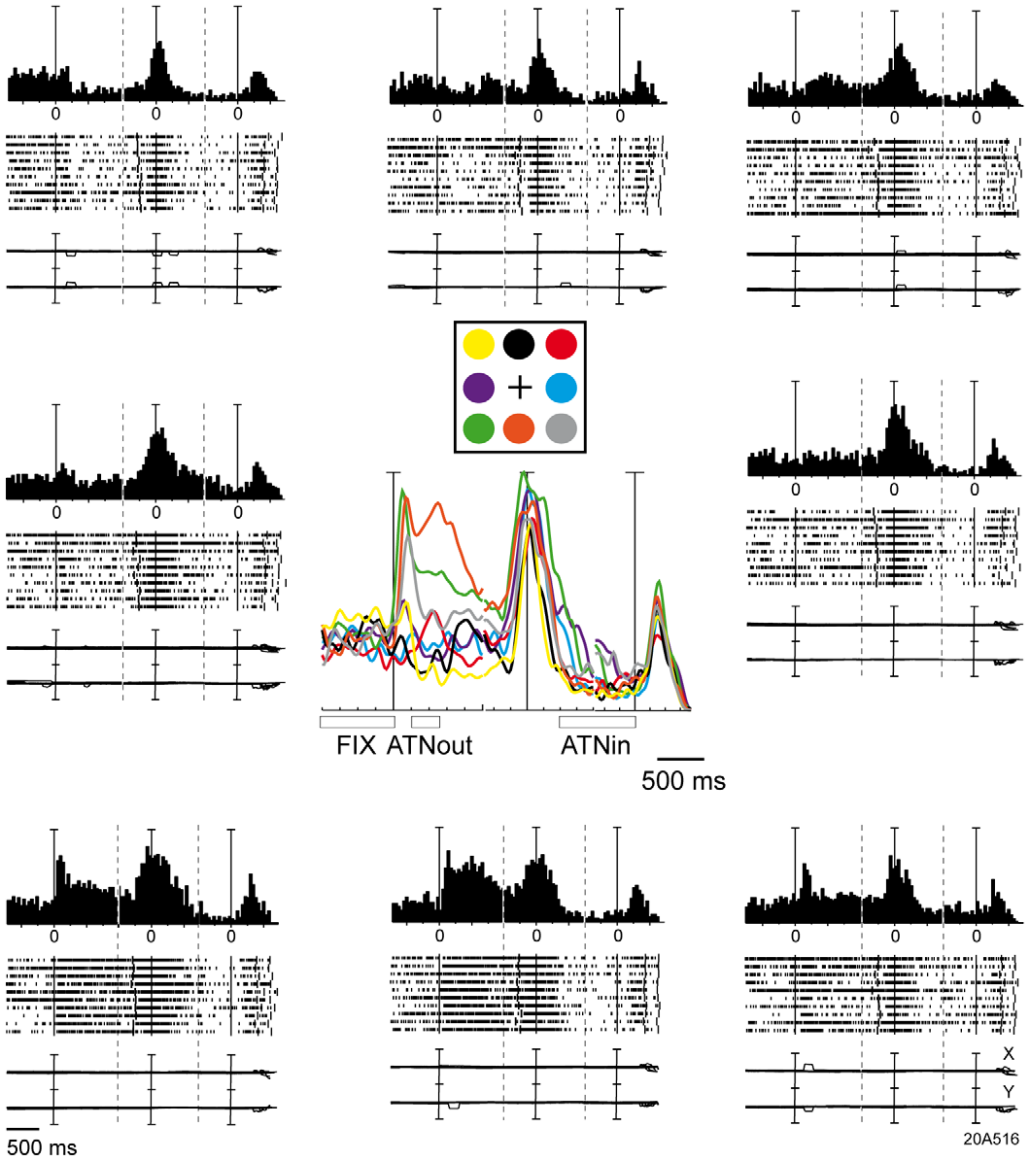
Example of a cell modulated during outward and inward attention epochs. This cell was excited during outward attention epoch when attention was covertly directed towards bottom locations, and inhibited during inward attention epoch for all attended locations. In addition, this cell was excited during button release and in the visual epoch, especially in the 3 lower positions. Neural activity and eye traces are aligned three times: from left to right: with the cue onset, with the button release and with the change in color of the fixation point. Peri-event time histograms: binwidth, 40 ms; scalebars, 180 spikes/s. Eyetraces: scalebar, 60°. Other details as in Figures 1 and 2.

Selective responses in the different task epochs could be found in combination in individual neurons: 31 cells were driven by both outward and inward shifts of attention, as the example reported in Figure 7. This is a cell whose activity was strongly modulated by the covert shift of attention towards the cue (outward attention epoch), but also by the action of button press, and by the bringing back of attention focus towards the fixation point (inward attention epoch). This last modulation was actually an inhibition. A one-way ANOVA on the activity of this cell around the button press (from 150 ms to 650 ms after target onset) gave a significant influence of target position (p<0.05). Therefore, the example of Figure 7 shows that the effect of attention can modulate not only the ongoing activity but also the motor-related activity of a single cell. The large majority of V6A cells are of this type.

**Figure 8:**
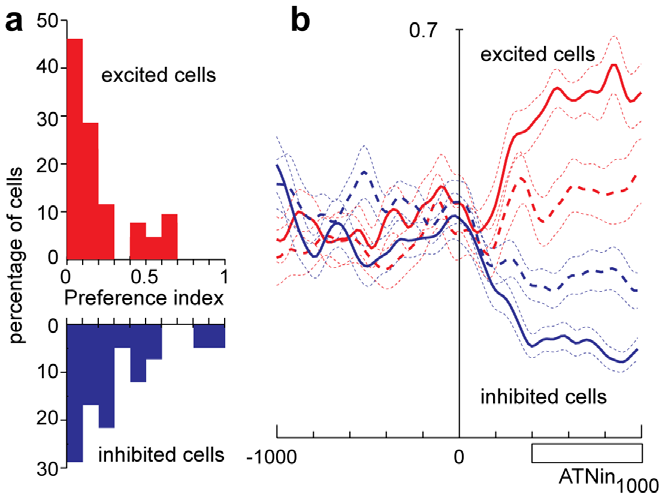
Activity modulation during inward attention epoch. **a**) Distribution of preference indices (see Experimental procedures) of cells excited (red histogram) and inhibited (blue histogram) during inward attention epoch. **b**) Effect of the increase of the level of attention at the fixation point on the neuronal population activity of V6A cells excited (red lines) or inhibited (blue lines) during inward attention epoch. The average SDF for the cue location evoking the maximal (excited cells) or minimal (inhibited cells) activity and the activity for the opposite cue locations are shown as continuous and dashed lines, respectively. The activity of cells in each population is aligned on the button release. Scale in abscissa: 200 ms/division; vertical scale 0.7. Other details as in Figures. 1 and 4.

Spatial tuning for inward attention epoch was less common than for outward attention epoch (17/63, 27%; 1-way ANOVA p<0,05). We calculated the distribution of preference indices separately for the population of excited and inhibited cells. The majority of excited cells (15/21, 71%) showed weak directional selectivity, with PI lower than 0.2 (Fig. 8**a**, red histogram). The directional selectivity of cells inhibited during inward attention epoch (Fig. 8**a**, blue histogram) was slightly higher than that of excited cells.

Figure 8**b** shows the population activity of the cells significantly excited (red lines) or inhibited (blue lines) during inward attention epoch (N = 21 and 42, respectively). The plots have been aligned on the button release. On average, cell activity changes after the button release, i.e, at a time when attention is redirected to the fixation point in order to detect its upcoming change in color. Cell activity then remained high or low (according to the type of cell) up to the end of the trial. This behavior is in line with a shift of attention to the fixation point. It is unlikely that it can be explained by visual stimulation, oculomotor, or any other motor-related activity, since no visual stimulation or motor behavior occured in that period and visual responses to the cue as well as motor responses from the button press are unlikely to occur so late and to last for such a long time. The delay of the change in cell discharge is longer than that observed in outward attention epoch (see Fig. 4**b**), in agreement with the view that the phenomenon is endogenously driven.

## Discussion

We have recorded responses of cells in monkey area V6A in a task that required covert attention shifts from a central fixation point outward to a peripheral location, and then inward shifts of attention back to the fixation point. The outward shift was exogenously driven by a visual cue while the inward shift was endogenously driven by the learned requirements of the task.

We found that the activity of 30% of V6A cells was modulated by the outward shift of covert attention, often in a direction-selective way, with half of the cells excited and half inhibited by the attentional shift. The onset and duration of attentional response correspond well to the typical temporal profile of exogenous attention shifts in humans [1] and to the attentional benefits on reaction times in the monkeys subject of Fig. 1**b**. Because the outward attention shift is driven exogenously by the visual cue signal, the cell response may contain a visual component. However, the latency and duration of attentional responses are clearly different from the typical visual responses in V6A (see Fig. 3). Visual responses have short latency, small variability between trials, and a duration that matches the duration of the stimulus [see also 26]. Attentional responses have longer latency and higher variability (see for instance rasters of spikes in the bottom part of Fig. 2). In cases where both visual and attentional responses were present in the same cell (e.g. in the bottom insets of Fig. 7), the brief visual response (same duration as the stimulus) was sometimes seen alone (e.g. in the bottom right panel), while in other cases (e.g. in the bottom central and left panels) it was followed by a tonic (attentional) discharge lasting hundreds of ms after the end of visual stimulation.

The activity of about 35% of V6A cells (63/182) was modulated by inward shifts of attention (inward attention epoch). The majority of the affected cells (about two-thirds) were inhibited, one-third were excited. These activity modulations were usually not spatially tuned, that is they did not vary significantly with the change in location of the cue. This was in agreement with the fact that during inward attention epoch the attention was focused on the same spatial location (the fixation point) regardless of cue location. It is worthwhile to note that contrary to outward shifts, inward shifts were not driven by an exogenous cue at the fixation point but rather instructed via the task demands, which required the detection of a color change at the fixation point after the peripheral flash. The inward shift of attention is thus endogenously driven, even thought the flash might have served as a trigger.

Activity modulations during outward and inward attention epochs may reflect a process representing the spatial location of the focus of attention. The spatial sensitivity of many cells is in line with this view. The excitation observed in the majority of neurons after outward attention shifts might reflect the better responsiveness at the new cued location commonly found in attentional studies. The inhibition observed in the majority of neurons when attention was directed back to the fixation point might reflect the decreasing responsiveness at the formerly cued location. Inhibition at previously cued locations is a common finding in attention research [27], [28] and an important contribution to the shaping of the ‘attentional landscape’. Comparison of the population activities in the outward and inward attention cases (Figs. 4 and 8) shows that the magnitude of the modulation is higher in the inward cases. This could be because in inward cases gaze and attentional focus are aligned, or because the inward attention shift is an endogenous process whereas the outward shift is exogenously driven. It is also possible that the modulation in the outward attention cases is smaller because attention is not maintained at the outward locus long enough to reach the same level of modulation as in the inward case.

It may be argued that the responses observed during the outward and/or inward attention epochs could be related to other cognitive processes, such as the preparation of the monkey to get ready for the button release/press, or arousal, or also the expectation of a later reward. Nevertheless, we believe that, if this were the case, we would have no spatial tuning of the responses, because the arm actions are button presses that occurred in a fixed spatial location. Since many cells here are spatially tuned in their attentional shifts, we believe we can rule out other interpretations of the results.

Many studies have focused on the influence of attention on neural activity in different brain areas, namely area LIP [4], [18], [29], [30], [31], [32], [33], superior colliculus [15], [34], frontal eye fields [30], [35], area 7a [36], [37], [38], [39], [40], area DP [40], area MT [29], [41], area VIP [41]. While a large amount of those studies shows that spatial attention modulates the neuronal response to a stimulus [see 6 for reviews], [ see 42], our findings provide evidence that spatial attention modulates the ongoing activity of a neuron, and this happens in an area never studied before in the attentional context. Other previous studies have demonstrated that the ongoing activity of cells in a high number of cortical areas, including V6A, is modulated by the direction of gaze [see 22], [43]. This was generally interpreted as an oculomotor effect. However, since the direction of gaze and the spotlight of attention are usually aligned, the gaze modulation could be the result of an attentional process which modulates the neuronal activity, rather than a direct oculomotor effect. By disengaging the attention from the point of fixation we have shown that this is the case for at least 30% of the neurons in area V6A (outward attentional effect). For these neurons, neural modulation was still present when covert attention was shifted without any concurrent shift of gaze direction, confirming that the modulating factor is the attentional process.

Recent brain imaging studies have shown that in the human medial superior parietal lobe there were transient activations by shifts of covert attention from one peripheral location to another [44], [45]. The activation was located in the anterior bank of the dorsalmost part of the parieto-occipital sulcus, that is just in front of where area V6 is located in human [46]. Since in macaque, area V6A is located just in front of area V6, in the anterior bank of the parieto-occipital sulcus, we suggest that the medial superior parietal region described by Chiu and Yantis [44] is the human counterpart of the macaque area V6A. If this were the case, we could conclude that in both macaque and human, area V6A is modulated by covert shifts of attention.

### Why an attentional modulation in a reaching area?

V6A is an area that contains visual, gaze, and arm movement-related neurons [21]. Present results show that V6A neurons are also modulated by covert spatial shifts of attention, and that visual, motor, and attentional responses can co-occur in single V6A cells. We had previously demonstrated that several single V6A cells were particularly sensitive to arm movements directed towards non-foveated objects [47]. The covert attentional modulations could allow these cells to select the goal of reaching during movement preparation, as well as to maintain encoded, and possibly to update, the spatial coordinates of the object to be reached out during movement execution.

Our results have shown a homogeneous spatial tuning of attention. This behavior parallels the homogeneous distribution of preferred gaze and reach directions observed in area V6A [22], [48], while it is in contrast with the preferred contralateral representation of the visual field, since the distribution of visual receptive fields in V6A mainly represents the contralateral visual field [23] (see also Fig. 5). In other words, the spatial tuning of attentional preference does not follow the sensory tuning, but rather the oculomotor and arm-reaching tuning found in V6A.

We believe that present results provide crucial support for the hypothesis that spatially-directed attention is linked to motor programming. Our study thus extends previous findings of a connection between attention and eye movement control [13], [14], [15], [16], [17], [31] to the case of reaching control, and points towards a neural substrate for interactions between attention and reaching that are known from human behavioral data [19], [20].

## Materials and Methods

### Experimental procedures

Experiments were carried out in accordance with National laws on care and use of laboratory animals and with the European Communities Council Directive of 24th November 1986 (86/609/EEC), and were approved by the Bioethical Committee of the University of Bologna and authorised by Ministero della Salute (Permit N° DM 47/2008-B, 6/4/2008, signed by the Direttore of the Dipartimento Sanità Pubblica Veterinaria). In accordance with the European Legislation and Guidelines and with the recommendations of the Wheatherall report, “The Use of non-human primates in research”, many measures were taken to ameliorate animal welfare: monkey training adopted positive reinforcement techniques. No deprivation, punishment, or suffering was inflicted. All procedures used have been approved and controlled by the Central Veterinary Service of the University of Bologna. Monkey food and water intake, as well as daily weight, were controlled by researchers and veterinarians, in order to monitor the wellbeing of the monkeys. Veterinarians were ready to detect, if present, clinical signs of pain or distress and to suggest the appropriate measures to increase animal welfare.

Three trained Macaca fascicularis of 6, 5 and 4 kg (Monkey L, Monkey C and Monkey X) sat in a primate chair and performed an attentional task with their head restrained. We performed single microelectrode penetrations using home-made glass-coated metal microelectrodes with a tip impedance of 0.8-2 MOhms at 1 KHz, and multiple electrode penetrations using a 5 channel multielectrode recording minimatrix (Thomas Recording, GMbH, Giessen, Germany). The electrode signals were amplified (at a gain of 10,000) and filtered (bandpass between 0.5 and 5 kHz). Action potentials in each channel were isolated with a dual time-amplitude window discriminator (DDIS-1, Bak electronics, Mount Airy, MD, USA) or with a waveform discriminator (Multi Spike Detector, Alpha Omega Engineering, Nazareth, Israel). Spikes were sampled at 100 KHz and eye position was simultaneously recorded at 500 Hz. Eye position was recorded using an infrared oculometer (Dr. Bouis, Karlsruhe, Germany) and was controlled by an electronic window (5×5 degrees) centered on the fixation target. Behavioral events were recorded with a resolution of 1 ms. We performed extracellular recordings on all the 3 animals; on two of them we also performed behavioral recordings.

Surgery to implant the recording apparatus was performed in asepsis and under general anesthesia (sodium thiopenthal, 8 mg/kg/h, i.v.). Adequate measures were taken to minimize the animal's pain or discomfort. Specifically, analgesics were used postoperatively (ketorolac trometazyn, 1 mg/kg i.m. immediately after surgery, and 1.6 mg/kg i.m. on the following days). Extracellular recording techniques and procedures to reconstruct microelectrode penetrations were similar to those described in other reports [22].

### The attentional task

Data were collected while monkeys were performing a task specifically designed to study the effect of covert spatial displacements of the spotlight of attention on neural responses. The monkeys sat in front of a fronto-parallel panel which was located 14 cm from the animal's eyes. The panel contained 3 green/red light emitting diode (LED; 4 mm in diameter; 1.6° of visual angle) that served as fixation point and target to be detected. The fixation point was the green/red LED located in the straight-ahead position. Two circular rings (12 mm in diameter; 4.8° of visual angle), illuminated by a yellow LED, served as a cue that indicated the spatial position of the subsequent target to be detected. The cue and target LEDs were located 15° peripherally on opposite sides from the fixation point.

The time sequence of the task is shown in Figure 1**a**. A trial began when the monkey decided to press the home-button near its chest. After pressing the button, the animal waited for instructions in complete darkness. It was free to look around and was not required to perform any action. After 1000 ms, the fixation LED lit up green. The monkey was required to look at the fixation target and to maintain the button press while waiting for an instructional cue.

After 1700–2200 ms, another LED (the CUE) lit up for 30–150 ms in one out of the two peripheral positions located 15° apart from the fixation point. After 1000–1500 ms a red flash (TARGET) of 5 ms occurred in the cued position. The monkey had to release the home-button as soon as it detected the target. The maximum time allowed to release the button was 1000 ms. If the monkey did not release the button during this period the trial was marked as error trial. After 1000–1500 ms, the fixation point changed in color from green to red. The monkey had to press the home-button again (maximum time to press was 1000 ms) to drink the reward. Home-button pressing ended the trial, issued monkey reward, and started the next trial.

The correctness of the animal's performance was evaluated by a software supervisor system [49] which checked the status of microswitch (monopolar microswitches, RS components, UK) mounted under the home-button. Button presses/releases were checked with 1 ms resolution.

Displacements of the spotlight of attention towards the two peripheral positions were typically tested as a randomized sequence in order to collect trials in one position intermingled with the other. Up to ten trials for each position were collected (20 trials in total). The panel could be rotated in 4 different positions (horizontal, vertical, and 2 oblique positions in between the two), allowing to test up to 8 spatial displacements of the spotlight of attention.

The task was performed in darkness. Eye fixation was always maintained in the straight ahead position within an electronic window of 5° amplitude. Fixation had to remain within this window throughout each trial until the fixation point switched off, otherwise the trial was aborted and a new one began without any reward. Off line inspection of eye records allowed to check for actual performance of fixation.

### Neuronal data analysis

We divided the trial into functional epochs, defined as follows (see bottom part of Figure 1**a**):

- FIX: steady fixation of the LED from its appearance to the cue onset; it contains the baseline activity of the neuron, used to compare the cell activity during the other epochs.
- VIS: from 40 to 150 ms after the cue onset; it could contain the passive visual response evoked by the cue appearance.
- outward attention epoch (ATNout): from 200 to 500 ms after the cue onset; it could contain the response due to the covert, peripheral displacement of the spotlight of attention.
- inward attention epoch (ATNin): from 400 ms after button release to the change in color of the fixation point; during this epoch the animal concentrates attention on the fixation point, as it has to detect the fixation point's change in color.

For behavioral analysis, the reaction time between target onset and button release was determined.

Only units which were tested in at least 7 trials for at least two target positions were included in the analysis. This is a conservative choice connected to the implicit high variability of biological responses [see 50 for detailed explanation].

For each neuron, the mean firing rate was calculated for each trial in outward attention epoch and inward attention epoch, and statistically compared with the mean firing rate in epoch FIX (two-tailed Student's t-test; significance level, p<0.02 with Bonferroni correction for multiple comparisons). This comparison was performed for each spatial location. Units with a significant discharge during at least one of the two attentional epochs were considered task related and were further analyzed. Excited cells during ATNout were defined as those cells whose discharge during ATNout was stronger than the one during FIX. Inhibited cells during ATNin were defined as those cells whose discharge during ATNin was stronger than the one during FIX. The same was done for the epoch ATNin.

The spatial tuning of activity in the task-related cells was analyzed statistically by comparing the mean firing rate in each target position (one-way ANOVA, F-test; significance level, p<0.05) for each of the functional epochs described above. A neuron was defined as 'spatially tuned' when it showed a statistically significant difference in mean firing rate in the same epoch in different spatial locations. Direction selectivity of neurons modulated during outward attention epoch and/or during inward attention epoch was quantified by a preference index (PI) for each functional epoch as follows:
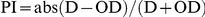
where D =  maximal discharge for cells excited with respect to FIX or minimal discharge for cells inhibited with respect to FIX, and OD =  discharge for the opposite position. PI ranged from 0 to 1.

Population activity of tested neurons was calculated as averaged spike density functions (SDFs). A SDF with a Gaussian kernel of half-width 40 ms was calculated for each neuron included in the analysis, averaged across all the trials for each tested condition, and normalized to the peak discharge of the neuron in the behavioral epochs of interest. The normalized SDFs were then averaged to derive population responses. We statistically compared the population SDFs with a permutation test with 10,000 iterations comparing the sum of squared errors of the actual and randomly permuted data.

### Behavioral data

We performed psychophysical measurements in separate sessions on 1 animal. In these sessions we collected reaction times of the monkey in valid trials, in which the target appeared in the cued position, and in invalid trials, in which the target appeared in the uncued position. These reaction times were recorded separately from the physiological data because the physiological recordings contained only valid trials. We recorded behavior during batteries of trials containing 20% of invalid trials randomly interleaved with valid trials. We tested two opposite target positions, to the right and to the left of the fixation point.

Various inter-stimulus-intervals (ISIs) were tested:, we used ISIs = 150 ms, 450 ms, 1000 ms (similar to the ISIs tested in Bowman et al., [25]). A repeated measures ANOVA (p<0.05) with factors: ISI (3 levels) and validity (2 levels) was used to assess the effect of validity, of ISI, and of the interaction between the two, on reaction time to target detection. To assess the validity effect for each ISI, post hoc comparisons using the Newman Keuls correction were used.

## References

[1] Posner MI (1980). Orienting of attention.. Q J Exp Psychol.

[2] von Helmholtz H (1867). Handbuch der Physiologischen Optik..

[3] Spitzer H, Desimone R, Moran J (1988). Increased attention enhances both behavioral and neuronal performance.. Science.

[4] Colby CL, Duhamel JR, Goldberg ME (1996). Visual, presaccadic, and cognitive activation of single neurons in monkey lateral intraparietal area.. J Neurophysiol.

[5] Connor CE, Preddie DC, Gallant JL, Van Essen DC (1997). Spatial attention effects in macaque area V4.. J Neurosci.

[6] Desimone R, Duncan J (1995). Neural mechanisms of selective visual attention.. Annu Rev Neurosci.

[7] Fischer B, Boch R (1985). Peripheral attention versus central fixation: modulation of the visual activity of prelunate cortical cells of the rhesus monkey.. Brain Res.

[8] Kodaka Y, Mikami A, Kubota K (1997). Neuronal activity in the frontal eye field of the monkey is modulated while attention is focused on to a stimulus in the peripheral visual field, irrespective of eye movement.. Neurosci Res.

[9] Awh E, Armstrong KM, Moore T (2006). Visual and oculomotor selection: links, causes and implications for spatial attention.. Trends Cogn Sci.

[10] Deubel H, Schneider WX (1996). Saccade target selection and object recognition: Evidence for a common attentional mechanism.. Vision Res.

[11] Hoffman JE, Subramaniam B (1995). The role of visual attention in saccadic eye movements.. Percept Psychophys.

[12] Kowler E, Anderson E, Dosher B, Blaser E (1995). The role of attention in the programming of saccades.. Vision Res.

[13] Cavanaugh J, Wurtz RH (2004). Subcortical modulation of attention counters change blindness.. J Neurosci.

[14] Hamker FH (2005). The reentry hypothesis: the putative interaction of the frontal eye field, ventrolateral prefrontal cortex, and areas V4, IT for attention and eye movement.. Cereb Cortex.

[15] Ignashchenkova A, Dicke PW, Haarmeier T, Thier P (2004). Neuron-specific contribution of the superior colliculus to overt and covert shifts of attention.. Nat Neurosci.

[16] Moore T, Armstrong KM, Fallah M (2003). Visuomotor origins of covert spatial attention.. Neuron.

[17] Thompson KG, Biscoe KL, Sato TR (2005). Neuronal basis of covert spatial attention in the frontal eye field.. J Neurosci.

[18] Lui Y, Yttri EA, Snyder LH (2010). Intention and attention: different functional roles for LIPd and LIPv.. Nat Neurosci.

[19] Castiello U (1996). Grasping a fruit: selection for action.. J Exp Psychol Hum Percept Perform.

[20] Deubel H, Schneider WX, Paproppa I (1998). Selective dorsal and ventral processing: Evidence for a common attentional mechanism in reaching and perception.. VisCogn.

[21] Galletti C, Kutz DF, Gamberini M, Breveglieri R, Fattori P (2003). Role of the medial parieto-occipital cortex in the control of reaching and grasping movements.. Exp Brain Res.

[22] Galletti C, Battaglini PP, Fattori P (1995). Eye position influence on the parieto-occipital area PO (V6) of the macaque monkey.. Eur J Neurosci.

[23] Galletti C, Fattori P, Kutz DF, Gamberini M (1999). Brain location and visual topography of cortical area V6A in the macaque monkey.. Eur J Neurosci.

[24] Luppino G, Hamed SB, Gamberini M, Matelli M, Galletti C (2005). Occipital (V6) and parietal (V6A) areas in the anterior wall of the parieto-occipital sulcus of the macaque: a cytoarchitectonic study.. Eur J Neurosci.

[25] Bowman EM, Brown VJ, Kertzman C, Schwarz U, Robinson DL (1993). Covert orienting of attention in macaques. I. Effects of behavioral context.. J Neurophysiol.

[26] Galletti C, Squatrito S, Maioli MG, Riva Sanseverino E (1979). Single unit responses to visual stimuli in cat cortical areas 17 and 18. II. Responses to stationary stimuli of variable duration.. Arch Ital Biol.

[27] Posner MI, Cohen Y, Bouma H, Bouwhuis D (1984). Components of visual orienting.. Attention and Performance: Erlbaum.

[28] Klein RM (2000). Inhibition of return.. Trends Cogn Sci.

[29] Herrington TM, Assad JA (2010). Temporal sequence of attentional modulation in the lateral intraparietal area and middle temporal area during rapid covert shifts of attention.. J Neurosci.

[30] Buschman TJ, Miller EK (2007). Top-down versus bottom-up control of attention in the prefrontal and posterior parietal cortices.. Science.

[31] Bisley JW, Goldberg ME (2010). Attention, Intention, and Priority in the Parietal Lobe.. Annu Rev Neurosci epub ahead of print.

[32] Goldberg ME, Bisley JW, Powell KD, Gottlieb J (2006). Saccades, salience and attention: the role of the lateral intraparietal area in visual behavior.. Prog Brain Res.

[33] Gottlieb JP, Kusunoki M, Goldberg ME (1998). The representation of visual salience in monkey parietal cortex.. Nature.

[34] Muller JR, Philiastides MG, Newsome WT (2005). Microstimulation of the superior colliculus focuses attention without moving the eyes.. PNAS.

[35] Wardak C, Ibos G, Duhamel JR, Olivier E (2006). Contribution of the monkey frontal eye field to covert visual attention.. J Neurosci.

[36] Rawley JB, Constantinidis C (2010). Effects of task and coordinate frame of attention in area 7a of the primate posterior parietal cortex.. J Vis.

[37] Bushnell MC, Goldberg ME, Robinson DL (1981). Behavioral enhancement of visual responses in monkey cerebral cortex. I. Modulation in posterior parietal cortex related to selective visual attention.. J Neurophysiol.

[38] Mountcastle VB, Pompeiano O, Marsan CA (1981). Functional properties of the light-sensitive neurons of the posterior parietal cortex and their regulation by state controls: Influence on excitability of interested fixation and the angle of gaze.. Brain Mechanisms of Perceptual Awarness and Purposeful Behavior.

[39] Constantinidis C, Steinmetz MA (2001). Neuronal responses in area 7a to multiple-stimulus displays: I. neurons encode the location of the salient stimulus.. Cereb Cortex.

[40] Raffi M, Siegel RM (2005). Functional architecture of spatial attention in the parietal cortex of the behaving monkey.. J Neurosci.

[41] Cook EP, Maunsell JH (2002). Attentional modulation of behavioral performance and neuronal responses in middle temporal and ventral intraparietal areas of macaque monkey.. J Neurosci.

[42] Constantinidis C (2006). Posterior parietal mechanisms of visual attention.. Rev Neurosci.

[43] Bremmer F, Pouget A, Hoffmann KP (1998). Eye position encoding in the macaque posterior parietal cortex.. Eur J Neurosci.

[44] Chiu YC, Yantis S (2009). A domain-independent source of cognitive control for task sets: shifting spatial attention and switching categorization rules.. J Neurosci.

[45] Esterman M, Chiu Y, Tamber-Rosenau BJ, Yantis S (2009). Decoding cognitive control in human parietal cortex.. PNAS.

[46] Pitzalis S, Galletti C, Huang RS, Patria F, Committeri G (2006). Wide-field retinotopy defines human cortical visual area V6.. J Neurosci.

[47] Marzocchi N, Breveglieri R, Galletti C, Fattori P (2008). Reaching activity in parietal area V6A of macaque: eye influence on arm activity or retinocentric coding of reaching movements?. Eur J Neurosci.

[48] Fattori P, Kutz DF, Breveglieri R, Marzocchi N, Galletti C (2005). Spatial tuning of reaching activity in the medial parieto-occipital cortex (area V6A) of macaque monkey.. Eur J Neurosci.

[49] Kutz DF, Marzocchi N, Fattori P, Cavalcanti S, Galletti C (2005). Real-time supervisor system based on trinary logic to control experiments with behaving animals and humans. Journal of Neurophysiology 93: 3674-3686.. Epub 2005 Feb.

[50] Kutz DF, Fattori P, Gamberini M, Breveglieri R, Galletti C (2003). Early- and late-responding cells to saccadic eye movements in the cortical area V6A of macaque monkey.. Exp Brain Res.

